# Improve how science advice is provided to governments by learning from “experts in expert advice”

**DOI:** 10.1371/journal.pbio.3002004

**Published:** 2023-02-21

**Authors:** Roger Pielke

**Affiliations:** Department of Environmental Studies, University of Colorado Boulder, Boulder, Colorado, United States of America

## Abstract

The COVID-19 pandemic revealed weaknesses in how science advice is provided to governments. This Perspective suggests 5 practical steps that the scientific community can take to improve the practice of expert advisory mechanisms in an emergency.

## Teaser

During the Coronavirus Disease 2019 (COVID-19) pandemic, science advisory mechanisms did not always live up to expectations. What practical steps can the expert community take to improve advisory mechanisms in an emergency?

The first assessments of the global response to the COVID-19 pandemic suggest that overall, the world did not perform up to expectations [1,2]. Judging by the first reports from the Evaluation of Science Advice in a Pandemic Emergency (EScAPE) project [3–7], the same can be said about science advisory mechanisms.

The EScAPE project, which I am involved in, involves more than a hundred researchers across almost 20 countries and aims to document and evaluate the roles of official (and in some cases, unofficial) government science advisory mechanisms during the COVID-19 pandemic. To date, we have learned that formal government science advisory mechanisms performed unevenly across national and subnational settings. In fact, there is a lack of consensus on exactly what “science advice” is supposed to represent. In most countries around the world, political fault lines during the pandemic centered on possible trade-offs between health and the economy [1,2]. For some countries, science advice was construed narrowly, as in the United Kingdom, where a report by the UK parliament found that science advice emphasized public health guidance and neglected economics expertise [8]. In other countries, experience in other recent crises (such as the Fukushima nuclear crisis in Japan) meant that national science advisory mechanisms already had an increased capacity leading into the pandemic [6]. Still others, such as Italy, assembled a range of expert advisors across public health, social sciences, and ethics, but did not integrate the work of these disparate groups into coherent advice for policy makers [4]. And a few countries, like the United States, lacked formal mechanisms of high-level government science advice, relying instead on political appointees.

Another finding emerging from the EScAPE project is the importance of the rise of “shadow” science advisory mechanisms outside of government. These are groups of credentialed experts who self-organize to challenge government science advice or government policies. Notable examples include Independent SAGE in the UK and the Ventenskapsforum in Sweden. Shadow science advisory bodies often include legitimate expertise, but sometimes center on fringe views or those reflecting political agendas couched in the language of science.

Shadow science advice has always been present, especially in the context of highly political debates, but in the pandemic, shadow science advice took on new prominence, raising questions around its potentially pathological effects on legitimacy and trust in expertise. In other contexts, shadow science advice contributed positively to decision-making, such as in the Philippines [9]. A key lesson of the pandemic for the expert community is to develop a much more sophisticated understanding of the contexts in which their self-organization to challenge governments works to improve decision-making (e.g., such as motivating greater transparency or accountability) and when it makes decision-making more difficult (e.g., such as when it leads to a loss of legitimacy or trust).

How can science advisory mechanisms be made fit for purpose? Five lessons for improving government science advice have already emerged from examining performance during the COVID-19 pandemic.

1. Governments should put in place civil servants who have career expertise in working at the interface of expertise and decision-making. A model for this can be found in the practice of diplomacy, where so-called sherpas and sous-sherpas work tirelessly behind the scenes to make it possible for diplomacy to work. The effective provision of science advice is no less complicated or important than diplomacy and requires being treated similarly as a profession to support the work of both experts and policy makers.
2. Across the world, we have seen a diverse range of expectations in what is required of government science advisors. Are they expected to be on call to answer questions posed by policy makers? Are they to ask and answer questions themselves? To provide policy advice? If so, to advocate a “best” course of action or to serve as an “honest broker,” providing a set of options for policy makers to consider? During the pandemic, such questions were infrequently asked or answered. A lack of shared expectations for the roles and responsibilities of science advisors should be addressed by creating clear and unambiguous terms of reference for their work.
3. In many places, the way in which expert advisory bodies are assembled has not been transparent and little clear justification has been provided for which experts were selected, holding what expertise, and to serve what purposes. This is one area where career professionals (see #1) can lay the groundwork for inviting experts to serve as advisors long before an emergency situation occurs. Each country could compile and keep updated a list of subject-matter experts to consult when an emergency develops, based on the nature of advice that is desired (see #2). A sufficient diversity of disciplines, orientations, characteristics, and political or policy views would be important to include in an expert advisory panel, depending on its role (see #2).
4. As the pandemic unfolded, in many countries it became clear that expert advisors were often unsure of the expectations of the role when it came to communicating with the public, especially through the media. Did they have a responsibility to echo consensus positions of their committee or panel? Were they instead to share the personal views they had argued before their advisory body colleagues? Perhaps it was to express support for or advice against government policies as they saw fit? Experts on topics as diverse as military intelligence and central banking have long-established norms of conduct and communication that could inform the development of similar norms and practices across expert advisory mechanisms when an emergency occurs [10,11]. Experts should receive training for dealing with the media, as learning on the job can be difficult.
5. Expertise is only useful in decision-making if political leaders respect advice. In governmental decision-making, advisors advise and decision-makers decide. While real-world practices are often messier and colored in shades of gray, democratic accountability always resides with those who are accountable to the public. That means that elected officials should publicly support the importance of advice even when they choose not to accept it, which is of course their right. However, the public deserves to know what advice is given and the justification leaders have for following it, or not. Political leadership entails supporting the practice of advice to foster legitimacy and trust among the broader public.

In general, science advisory mechanisms function well on topics such as vaccine approval, climate change, biodiversity, and post-disaster assessments. However, government science advisory mechanisms generally were not prepared for the scope and depth of the COVID-19 pandemic emergency, which may have contributed to larger failures in pandemic responses [1,2]. Going forward, it will be important to further develop institutions of formal science advice. Such institutions must go well beyond organizational charts to include the development of a professional class of “experts in expert advice” who can support work at the intersection of science and policy, as well as the creation of a new set of norms and expectations for the importance and legitimacy of expert advice. The future is sure to bring new pandemics and other emergencies that we have not even considered yet. The time to prepare is now.
